# A Comparative Study of Behavior Problems among Left-Behind Children, Migrant Children and Local Children

**DOI:** 10.3390/ijerph15040655

**Published:** 2018-04-01

**Authors:** Hongwei Hu, Jiamin Gao, Haochen Jiang, Haixia Jiang, Shaoyun Guo, Kun Chen, Kaili Jin, Yingying Qi

**Affiliations:** 1 School of Public Administration and Policy, Renmin University of China, No. 59, Zhongguancun Street, Haidian District, Beijing 100872, China; whuhhw@126.com; 2 Institute of Population Research, Peking University, No. 5, Yiheyuan Road, Haidian District, Beijing 100871, China; hnjhx@163.com; 3 School of Humanities and Social Sciences, North China Electric Power University, No. 689, Huadian Road, Lianchi District, Baoding 071003, China; gsy750086@126.com (S.G.); ckxs0514@126.com (K.C.); jinkaili1997@126.com (K.J.); yy20150918@126.com (Y.Q.)

**Keywords:** behavioral problems, left-behind children, migrant children

## Abstract

This study aims to estimate the prevalence of behavioral problems among left-behind children, migrant children and local children in China, and to compare the risks of behavioral problems among the three types of children. Data on 4479 children aged 6–16 used in this study were from a survey conducted in China in 2017. The school-age version of the Children Behavior Checklist was used to measure children’s behavioral problems. Descriptive analysis, correlation analysis, and logistic regressions were conducted. The prevalence of behavioral problems was 18.80% and 13.59% for left-behind children and migrant children, respectively, both of which were higher than that of local children. Logistic regression analysis showed that after adjustments for individual and environmental variables, the likelihood of total, internalizing and externalizing behavior problems for left-behind children and migrant children were higher than those for local children; left-behind children had a higher likelihood of internalizing problems than externalizing problems, while migrant children had a higher prevalence of externalizing problems. Left-behind children had a higher prevalence of each specific syndrome than migrant and local children. Both individual and environmental factors were associated with child behavioral problems, and family migration may contribute to the increased risks. Left-behind and migrant children were more vulnerable than local children to behavioral problems.

## 1. Introduction

Children’s behavioral problems, which are reflections of children’s psychological wellbeing, have become a major concern in children’s development and functioning across the world. Studies reported that approximately 20% of global children were affected by behavioral problems in 2011 [1]. Behavioral problems of children may result in serious disruption of the educational process, self-injury or mental health problems [2,3,4].

Studies showed that children’s emotional and behavioral problems were associated with broad factors being constructed as a hierarchical system. Children’s personal characteristics and environmental factors, including family, school and living surroundings were related to the types, causes and patterns of the emotional and behavioral problems. Tehrani, et al. (2011) provided evidence that girls had more probability of aggressive behaviors and attention problems than boys [5]. Children in poor physical health conditions were more likely to experience bipolar disorders [6]. A body of research indicated that family environment had leading influences on children’s emotional and behavioral problems. Family structures and circumstances are strongly associated with children’s development. Studies showed that children who lived in the kinship foster care system (grandparents or other family members were children’s caregivers) [7], lived with parents who conflicted frequently [8], lived in a poor household or lacked parental concern had more emotional and behavioral problems [9]. For middle-childhood children and adolescents, schools become another circumstance that had direct effects on children’s development. Peer relationship and school circumstances (i.e., school faculty and resources) are essential to children’s emotional and behavioral well-beings. Peer rejection may give rise to children’s antisocial behaviors and those children who were popular among peers were less likely to be aggressive and had less trouble in dealing with social problems [10]. Studies also provided evidence that children’s living conditions had an impact on their emotional and behavioral disorders. Children living in urban slums or in rural areas had higher risks of emotional and behavioral problems due to social deprivation [11].

The prevalence of children’s behavioral problems in China was 12.97% among children aged 4–16 in 1992 [12]. Since the early 1990s, China has been undergoing rapid industrialization and urbanization, yielding a large scale of migrant and left-behind children. According to the sixth national population census, the number of left-behind children whose parent(s) was a migrant had reached 61.02 million in China in 2010, while migrant children had reached 35.81 million [13]. Unlike the traditional nuclear or joint family, the left-behind family is always characterized by the absence of either one or both parents, leaving the children guarded by grandparents, a single parent or other family members. For migrant children, migration with parents means adaptation to new environments, which may increase the probability of children’s emotional and behavioral disorders. Previous studies in China indicated that left-behind children were more likely to be anxious, depressed, as well as to have somatic complaints and hostile behaviors, while migrant children tended to have difficulty in social problems and being depressed [14,15].

However, previous studies had several limitations. First, the number of studies regarding emotional and behavioral problems of the left-behind or migrant children in China was limited. Second, insufficient studies focused on the comparison of emotional and behavioral problems among left-behind, migrant and local children. Third, the sample sizes in previous studies were small. Last but not the least, most of previous literature failed to analyze the associated factors of emotional and behavioral problems in a multi-level perspective, resulting in an overemphasis on a specific dimension other than a whole system.

This present study aims to answer two research questions. First, it examines the prevalence of total, internalizing and externalizing emotional and behavioral problems as well as the prevalence of each specific syndrome among left-behind, migrant and local children. Second, given the potential impacts of individual characteristics and environmental factors on child psychological health, this study investigates the association between multi-level risk factors and children’s behavioral problems. This study aims to fill the gaps in this topic for China and contribute to the world literature in the context of countries with a large scale of migration.

## 2. Methods

### 2.1. Data

Data were obtained from a survey conducted in Chongqing, one typical city with a large scale of immigration and emigration in China, between September and October 2017 with a focus on psychological and behavioral outcomes of different types of children. This survey was carried out by the Chongqing Education Bureau and other educational institutions. The survey had a stratified, multi-stage systematic selection design for sample selection. A total number of 30 schools, including elementary and middle schools from 9 counties, were selected. Within each school, one class was selected randomly from each grade. Investigators interviewed the parents or guardians of the children who studied in selected classes based on the survey questionnaires. The original selected sample included parents or guardians of 4500 children, and 4479 with complete information on key variables were used in this study. All participants were required to fill out the questionnaires that contained information on children’s socio-demographic characteristics, health conditions, parental and family information, school situation as well as psychological and behavioral conditions.

### 2.2. Child Behavioral Screening

The school-age version of the Achenbach Child Behavior Checklist (CBCL/6–18) in Chinese was used in this survey by instructing parents or guardians who were familiar with the children to report the children’s behavioral problems. The school-age version of CBCL is standardized well and has satisfactory psychometric properties [16]. The Achenbach Child Behavior Checklist was introduced to China in 1980s, and was modified to be adapted to Chinese culture and actual conditions by the Shanghai Mental Health Center, which resulted in the difference between Chinese and English versions. The Chinese version of the CBCL applied in this study was generally consistent with the English version published in 1983, in which the measured syndromes varied in different age and gender groups (16 standard syndromes in all). The current English version CBCL used in Western countries was the revised version published in 1991, which reclassified into 9 standard syndromes that can be applicable in all age and gender groups. Detailed information of the measured syndromes was presented in Table 1.

In this study, the school-age version checklists included 113 symptoms to identify the children’s behavioral problems, and the 113 specific items and the specific contents under each item were basically the same between the Chinese and the current English version CBCL. Each symptom question is scored 0, 1, 2, referring to no such symptom, sometimes having this symptom and often having this symptom, respectively. The CBCL yields specific syndrome scores and two broadband scores (internalizing problems and externalizing problems) by adding up the scores of specific symptoms under each syndrome. The scores of total behavioral problems were calculated by the sum of the scores of all symptoms.

We calculated raw scores of specific syndromes, which were used to compare with scores of the norm sample. Children whose raw scores are above the upper threshold of the norm sample in at least one syndrome were considered to have behavioral disorders. The scores of the norm sample applied in this study were from an epidemiology survey on children’s psychological and behavioral problems, which was conducted among 24,013 children from 22 cities in 1992 with national representativeness. The survey was carried out by the Shanghai Mental Health Center and 25 other institutions based on the 1983 English version of the CBCL. The nationwide scores of the norm sample have not been updated since 1992. It was proved to have validity and reliability in China and has been widely used in research on Chinese children’s behavioral problems for the past decades [12]. Because the measured syndromes differed by age and gender groups according to the norm sample, we calculated and compared the syndrome scores separately.

Internalizing problems focus on children’s emotional disturbances including schizoid or depressed, social-withdrawal and somatic complaints. Externalizing problems refer to behavioral deficits, such as aggressive, delinquent, etc. The raw scores for internalizing and externalizing problems were calculated, and scores above the 90th percentile were considered clinically significant [17]. Table 1 lists how the syndromes we measured and the syndrome scores of norm sample differed by gender and age.

### 2.3. Variables

The outcome variable was a binary variable: whether the child had behavioral disorders. Children’s behavioral problems were reported by their parents or guardians in response to the checklist based on the school-age version of the CBCL.

Child category was employed as the main independent variable in this study. Local children in this survey were defined as children who were registered in Chongqing’s hukou system and living with their parents. Migrant children were defined as children who immigrated with parents from other places, lived but did not register in Chongqing. The left-behind children were registered and living in Chongqing but their parent(s) migrated to work elsewhere for over 6 months.

Control variables in this study included individual characteristics and circumstances such as family, school and residence factors following previous studies [19,20]. Personal characteristics variables consisted of gender (0 = female and 1 = male), age (0 = 6–11 and 1 = 12–16), health conditions (0 = good physical health and 1 = fair physical health) and whether they were the only child in a family (0 = no and 1 = yes). Family and household factors contained household income level (0 = high, 1 = medium and 2 = low), parents’ marital status (0 = unmarried and 1 = married), family conflicts (0 = frequent and 1 = seldom), parental alcoholic (0 = no and 1 = yes), maternal education attainment (0 = primary school and below and 1 = middle school and above), parent neglect children frequently in the past 6 months (0 = no and 1 = yes). We selected model school (schools with better faculty and facilities in specific district, 0 = no and 1 = yes) and whether children were bullied in school (0 = no and 1 = yes) as measurements for children’s school situations. Residence as a general measure of children’s living conditions was classified as “0 = urban area” and “1 = rural area”.

### 2.4. Statistical Analyses

The prevalence of behavioral problems among migrant, left-behind and local children was presented in the current study. Multivariable logistic regression analysis was employed to compare the risks of behavioral problems among the three types of children. The software STATA 13.0 for windows (Stata Corp, College Station, TX, USA) was utilized for statistical analysis.

Although the endogeneity of migration may be an obstacle in this study, we did not employ specific approaches to solve this endogeneity problem. Parental migration is likely to be influenced by a whole host of unobservable factors that may also affect children’s outcomes directly, leading to the endogeneity problem which may cause a possible estimation bias to some extent in this study [21,22]. However, it was acceptable and reasonable not to be too concerned about or to solve the endogeneity problems in our study for the following two reasons: on the one hand, there was a lack of necessary and exogenous variables concerning parents and family situations in the second-hand data; on the other hand, this study emphasized more children’s current behavioral problems after parental migration, rather than the causality of parental migration to children’s behavioral problems [23,24].

## 3. Results

### 3.1. Sample Description

Table 2 presents the characteristics of the study sample. In this study, 23.48% were left-behind children, and 34.46% were migrant children. 57.74% of children were between 6–11 years old, 46.50% were girls, 58.59% had good physical health, 57.11% were the only child in their families and 54.57% lived in urban area. Most children lived in a family with medium or poor household income (91.19%), with parents who were married (91.08%), with rare family conflicts (93.89%) and maternal education of middle school and above (76.83%). Moreover, 85.79% children did not suffer frequent parental neglect in the past 6 months. For the aspect of school, 91.28% of children studied in non-model schools and were not bullied by peers (91.93%).

### 3.2. Child Behavioral Problems

Table 3 shows the prevalence of children with total, internalizing, and externalizing behavioral problems by children group. Of these, 12.80% of children had behavioral problems, among which 9.55% and 9.52% had internalizing and externalizing problems, respectively. The left-behind children had the highest prevalence of total, internalizing and externalizing behavioral problems, with 18.80% for the total problem, and 15.19% and 13.20% for internalizing and externalizing problems. Migrant children had higher prevalence than local children for total and externalizing behavioral problems. For internalizing behavioral problems, although migrant children had higher prevalence than local children, the gap was relatively narrower.

### 3.3. Specific Syndromes for Child Behavioral Problems

The prevalence of specific syndromes on children’s behavioral problems differed by age and gender (see Table 4). For the boys aged 6–11, the category uncommunicative had the highest prevalence of 7.48%, followed by obsessive-compulsive, while for the girls in the same age group, schizoid-obsessive ranked as the leading behavioral problem, and in turn hyperactive. In the 12–16 age group, the prevalence of obsessive-compulsive, hostile withdrawal and somatic complaint were the top three of all specific syndromes in boys and, schizoid and cruel were the major behavioral problems in girls.

Correlations on child category and specific syndrome of behavioral problems appear in Table 4. The results showed that specific syndromes of behavioral problems differed significantly among the three child categories. Except for the hyperactive and social-withdrawal syndromes measured in boys aged 6–11, the left-behind children had higher prevalence of each behavioral problem syndrome than migrant and local children in all age and gender groups. In addition, migrant children’s prevalence was higher compared to local children in all specific behavioral problem syndromes except for schizoid in girls aged 12–16. The detailed prevalence of specific syndromes among different sub-groups is listed in Table 4.

### 3.4. Regression Results

Table 5 presents the adjusted odds ratios of total, internalizing and externalizing child behavioral problems in the multivariate regression models. Overall, after adjusting for personal characteristics and environmental factors, the child category was strongly correlated to children’s behavioral problems. The left-behind and migrant children had 2.00 times and 1.64 times higher risks of behavioral problems than local children, respectively. The left-behind children’s risk of having internalizing behavioral problems were 2.28 times higher than local children, while migrant children were 1.68 times higher. The left-behind children had higher risks of being exposed to internalizing behavioral problems, while migrant children were more likely to experience externalizing behavioral problems with 2.33 times higher risks than local children; and the left-behind children’s odds ratios for externalizing behavioral problems were 2.21.

Multivariable regression results indicated that by holding other variables constant, children’s physical health conditions were strongly associated with behavioral problems. Children who were in good health were less likely to have behavioral problems. In terms of household socioeconomic status, children with a medium or low level of household income had greater probability of having behavioral problems, including internalizing and externalizing disorders. In addition, maternal education attainment was related to children’s internalizing behavioral problems; a higher maternal education level may prevent children from behavioral disorders. Harmonious family relationship can protect children from both externalizing and internalizing behavioral problems. It was worth noting that children whose parent(s) drank constantly and neglected them frequently had more possibility of having behavioral problems. Children being bullied at schools had greater risks of internalizing behavioral problems, with odds ratios of 1.42, than those who did not, and had a 1.65 times higher likelihood of having total behavioral problems. Children living in rural areas were more likely to have total behavioral problems with odds ratios of 1.36, with 1.35 for internalizing behavioral problems.

Table 6 presents the adjusted odds ratios of the left-behind and migrant children for each syndrome on behavioral problems that were measured in the specific child group. The results showed that except for hyperactive, both the left-behind and migrant children were at higher risk than local children of experiencing each specific syndrome among boys aged 6–11. Migrant boys at this age had higher odds ratios for schizoid, uncommunicative, obsessive-compulsive, social-withdrawal, aggressive and delinquent syndromes. For girls at the same age, the left-behind children were statistically significant in all types of problematic behavioral syndromes. Although migrant children had higher risks of the majority of behavioral problem syndromes, there were no significant difference between migrant children and local children on social-withdrawal and sex problem syndromes. As can be seen in Table 6, migrant boys aged 12–16 were statistically significant only in aggressive syndrome compared to local children, but the left-behind children were more likely to have problems of schizoid, obsessive-compulsive, somatic complaints, hyperactive, aggressive, delinquent, hostile withdrawal and immature-hyperactive syndromes.

## 4. Discussion

This study used a school-based survey to investigate the prevalence of behavioral problems as well as each syndrome of emotional and behavioral well-beings among children, based on the application of the school-age version of the CBCL. In addition, this study measured the associated sociodemographic and environmental determinants of children’s behavioral problems.

We found that the prevalence of total behavioral problems of children aged 6–16 in Chongqing was 12.80%, which was different from previous studies and fell within the range of 8.04–25.80% in previous studies [25,26,27]. Among the three types of children, the left-behind children had the highest prevalence of total behavioral problems, internalizing and externalizing problems, followed by migrant children. In this study, the prevalence of reported total behavioral problems for the left-behind children was 18.80% which was less than a previous study that reported 41.3% in rural areas of Anhui province and similar to 18.0% in Ningxia province [28,29]. The prevalence for migrant children of total behavioral problems presented in this study was 13.59%, which was less than the 28.2% reported in a study conducted in Guangzhou [30].

This study suggests that left-behind children had higher risk of internalizing problems than externalizing problems, while migrant children had higher risk of externalizing problems than internalizing problems. Internalizing problems present as a cluster of problems within the self, such as depression and withdrawn, were different from externalizing problems being characterized by conflicts with the environment [31]. Family factors affect internalizing behavioral problems. A lack of parental care predicts more internalizing behavioral problems in children. For the left-behind children, most of them live in a kinship foster care system in which grandparents or other family members are their caregivers. First, the left-behind children cannot contact their parents regularly which may result in the absence of daily parental psychological care and guidance on children’s emotional health. Second, children growing up in a kinship foster care system tend to have more psychological distress and less family support, leading to behavioral problems both in internalizing and externalizing. The above two reasons may account for the higher prevalence of behavioral problems for left-behind children among the three types of children, and the higher prevalence of internalizing rather than externalizing problems among left-behind children. This study indicates that migrant children had a higher likelihood of externalizing problems. Jolande C. et al. (2003) suggested that environment accounted for 37% (while gene for 55%) of phenotypic stability for externalizing problems compared to 23% (while gene for 66%) for internalizing problems [32]. Studies have also argued that school and family environments had an impact on children’s externalizing behaviors [33], and parental negativity as well [34]. First of all, migration means a breakdown on the familiar and stable living environment. Migrant children have to reestablish their own social relationships and adapt to strange circumstances. This process may increase the likelihood of conflict with the environment. Second, quite a number of parents migrate more than once with their children. Frequent changes of environment imply that migrant children have to adapt to different schools and living surroundings. In addition, frequent mobility may give rise to parental negative emotions and stress which exert negativity on children. Family migration changes not only the family structure but also the care system and parent–child relationship, which may cause more challenges and worse psychological and health outcomes.

This study indicates that due to the changes of external circumstances, there were strong differences among the left-behind, migrant and local children in the risks of specific syndromes, except for the hyperactive category in boys aged 6–11. This may be because heritability accounted for about 76% of childhood hyperactivity disorder and environmental factors had little influence on its occurrence [35]. In this study, social-withdrawal and sex problems have no statistically significant difference between migrant and local girls aged 6–11, but were strongly correlated between the left-behind and local groups. Social-withdrawal of children can be presented as shyness, peer neglect, isolation and rejection, which is a complicated consequence constructed by an inhibited temperament, an insecure parent-child relationship, shared genetic vulnerabilities or traits with the parents, overly directive or protective parenting, peer rejection and the interaction among the listed reasons [36]. In this study, all of the left-behind children were registered in Chongqing and they studied together with the local children. Therefore, family factors, such as an insecure parent–child relationship, may be the key explanations for the difference of social-withdrawal. Besides, parents play vital roles in educating children with sexual and reproductive health knowledge. However, compared with children who live with their parents in migrant and local families, the left-behind children’s lack of parental guidance on sexual knowledge may increase their risks of sex problems. We also found that the significance of differences between migrant and local children disappeared in a majority of syndromes among girls and boys aged 12–16, which may suggest that the effects of family migration were declining. This may be because with the increase of age, adolescents have more social connections with friends and become less reliant on their family, which was consistent with previous studies [37].

Our study also shows that, except for gender and whether being the only child in a family, the remaining socio-demographic factors were correlated with either the total, internalizing or externalizing behavioral problems after controlling for other variables. Children aged 6–11, most of whom were in elementary schools, were predicted to have a higher likelihood of psychological and behavioral problems, which was consistent with previous studies [38]. Children with fair physical health conditions had higher risks of emotional and behavioral disorders, and children in poor physical health had almost three times the risk of total and externalizing behavioral problems, and 4.2 times for internalizing problems, which was in line with previous research [39].

Our study suggests that a better family environment was associated with lower risks of children’s emotional and behavioral well-beings. First, household socioeconomic status had effects on children’s mental and psychological health. Children with high household income levels or a more educated mother were less likely to have emotional and behavioral disorders [20,40]. This may be because limited financial resources and low social support from a low-income household are linked to high levels of parenting stress and harsh parenting practices. In addition, mothers with low socioeconomic status and low education were vulnerable to poor physical and mental health and more parenting stress [41]. Both harsh parenting, high parental stress and poor parental health conditions have been proved to be negative for a child’s psychological health [40]. Second, family relationships and parent-child relationships were correlated to children’s psychological and behavioral health. The findings of this study indicated that frequent family conflicts, unmarried parents and poor parent–child relationship (parents neglect their children frequently) did harm to children’s emotional and behavioral well-beings, which was in accordance with previous studies showing that the disruption of marital relationships and the absence of parental care increased the risks of children’s emotional and behavioral problems [42,43]. Besides, this study shows that parental alcoholism increased the probability of children’s unhealthy emotions and behaviors, indicating that unhealthy parental behaviors were associated with higher risks of children’s emotional and behavioral problems [44].

We observed that children who had experience of being bullied in schools had a higher likelihood of total and internalizing behavioral problems than those who did not. On the one hand, our findings verified that poor peer relationships may bring about adverse health outcomes. On the other hand, unlike the family relationship that had effects on both internalizing and externalizing aspects, peer relationships had greater effects on children’s internalizing emotional and behavioral problems, indicating that a peer relationship, as an acquired relationship from external circumstances, may trouble children more than conflicts with environments.

Last but not least, we found that residence was associated with children’s total and internalizing behavioral problems, showing that children who lived in rural areas had more risks of experiencing psychological and behavioral problems. This was in line with previous studies suggesting that poor housing environments or deprived areas exerted stress on child mental health and behavioral problems [45].

This study has a few limitations. First, the unsolved endogeneity problem about parental migration is an obstacle in this study, which may cause possible estimation bias to some extent. Parental migration is likely to be influenced by a whole host of unobservable factors, which may also have an influence on children’s behavioral problems directly. However, insufficient exogenous variables concerning family and school situation in the second-hand data limited us to using specific approaches to address the endogeneity problem in this study. Second, we collected information on children’s behavioral problems from guardians or parents, which may cause bias. There are three approaches to information collection on children’s behavioral problems, and information collected through the parents or guardians’ version of the CBCL has been proved to be more objective and comprehensive when compared with the child-report version or teacher-report version CBCL. Third, the scores of the nationwide norm sample used in this study were collected through an epidemiology survey in 1992. It may lead to limitations when we use it to assess children’s behavioral problems in current studies, as China has undergone dramatic development in the past decades. Although the scores of the nationwide norm sample of China have not been updated since 1992, it was the only standard score of norm sample with national representativeness and has been widely used as it has been considered to be scientific and applicable in current Chinese studies. Therefore, we have to admit this as one of our limitations.

Several strengths exist in this current study which may balance the limitations. First and foremost, we strictly applied the school-age CBCL version to measure the emotional and behavioral problems for children aged 6–16, and this is comprehensive and widely used. Second, a representative sample design was conducted in the survey which can capture the information of all types of children. Third, to our best knowledge, this is the first comparative study on children’s emotional and behavioral problems measured with the CBCL among left-behind, migrant and local children in China, and may be represented as one of the most comprehensive studies on this issue in LMIC (low and middle income countries) with large migrant populations. In addition, we measured the associated individual and environmental factors which are constructed as a multi-level system in terms of children’s behavioral problems. Notably, a cross-sectional design for this study cannot draw a causal association between personal characteristics and circumstantial factors with the outcome measures.

Last but not least, the findings that left-behind children and migrant children were at higher risks of behavioral problems have practical implications for policymakers in China. More attention and resources should be paid and allocated to the two vulnerable groups of children. Local government can play a vital role in this process by implementing specific polices to support the left-behind and migrant children, particularly in providing services on care and mental health. For schools, it is necessary to promote interaction and integration among different types of children, as well as to build up a network among teachers, parents and children to enhance communication.

## 5. Conclusions

This study showed a higher prevalence of internalizing and externalizing as well as specific syndromes of behavioral problems in left-behind children and migrant children than in local children. Personal characteristics including age and physical health conditions and circumstantial factors such as household income level, maternal education attainment, family and peer relationship were significantly associated with children’s emotional and behavioral well-beings. Family migration may exert essential impacts on children’s psychological health. Special attention ought to be paid to children’s behavioral problems, especially for vulnerable children suffering from family migration. More targeted policies are encouraged to help left-behind children engage in changed family environments and to promote the social integration of migrant children living in cities.

## Figures and Tables

**Table 1:** Differences in the specific measured items and related scores of the norm sample by gender and age.

	Aged 6–11		Aged 12–16	
	Boys	Girls	Boys	Girls
Syndromes (scores of norm sample)	Schizoid (6) Depressed (10) Uncommunicative (6) Obsessive-Compulsive (9) Somatic Complaints (7) Social-Withdrawal (6) Hyperactive (11) Aggressive (20) Delinquent (8)	Depressed (14) Social-Withdrawal (9) Somatic Complaints (9) Schizoid-Obsessive (4) Hyperactive (11) Sex problems (4) Delinquent (3) Aggressive (19) Cruel (4)	Somatic Complaints (11) Schizoid (8) Uncommunicative (15) Immature-Hyperactive (6) Obsessive-Compulsive (6) Hostile Withdrawal (11) Delinquent (9) Aggressive (19) Hyperactivity (10)	Anxious-Obsessive (18) Somatic Complaints (8) Schizoid (4) Depressed-Withdrawal (13) Immature-Hyperactive (12) Delinquent (12) Aggressive (18) Cruel (5)
Internalizing behavioral problem	Schizoid Depressed Uncommunicative Obsessive-Compulsive Somatic Complaints	Depressed Social-Withdrawal Somatic Complaints Schizoid-Obsessive	Somatic Complaints Schizoid Uncommunicative Immature-Hyperactive Obsessive-Compulsive	Anxious-Obsessive Somatic Complaints Schizoid Depressed-Withdrawal
Externalizing behavioral problem	Hyperactive Aggressive Delinquent	Hyperactive Sex problems Delinquent Aggressive Cruel	Delinquent Aggressive Hyperactive	Delinquent Aggressive Cruel

Notes: Scores of norm sample calculating for total problem and specific syndrome are in parentheses; “Schizoid” means social disorder which is in company with decreased interests and limited mobility; “Sex Problem” is characterized by sex-related problems: plays with sex parts too much, thinks about sex too much, and so on; all the detailed explanations of the 16 standard syndromes can be found in the Manual for the Child Behavior Checklist and Revised Child Behavior Profile (1983) [18].

**Table 2:** Characteristics of sample.

Characteristics	Percentage (%) (*n* = 4479)
Children’s category	
Local children	42.06
Left-behind children	23.48
Migrant children	34.46
Gender (reference: girls)	53.50
Age (reference: aged 6–11)	42.26
The only-child in family (reference: no)	57.11
Health conditions (reference: good physical health)	41.41
Household income level	
Good	8.81
Medium	62.91
Low	28.28
Parents’ marital status (reference: unmarried)	91.08
Family conflicts (reference: frequent conflicts)	93.89
Parental alcoholic (reference: no)	16.95
Maternal education attainment (reference: elementary school and below)	76.83
Parents frequently neglected children in the past 6 months (reference: no)	14.21
Studied in model school (reference: non-model school)	8.72
Children bullied in school (reference: no)	8.07
Residence (reference: urban areas)	45.43

**Table 3:** Prevalence of total, internalizing and externalizing behavioral problems among the left-behind, migrant and local children (%).

Scales	Total (*n* = 4479)	Left-Behind Children (*n* = 1053)	Migrant Children (*n* = 1545)	Local Children (*n* = 1881)	Chi2/F	*p*-Value
Total problem	12.80	18.80	13.59	8.80	61.879	0.000
Internalizing	9.55	15.19	9.77	6.20	63.410	0.000
Externalizing	9.52	13.20	11.65	5.73	56.196	0.000

**Table 4:** Prevalence of specific syndromes of behavioral problems among left-behind, migrant and local children (%).

Syndromes	Boys								Girls							
	Aged 6–11				Aged 12–16				Aged 6–11				Aged 12–16			
	Total Sub-Sample	Left-Behind	Migrant	Local	Total Sub-Sample	Left-Behind	Migrant	Local	Total Sub-Sample	Left-Behind	Migrant	Local	Total Sub-Sample	Left-Behind	Migrant	Local
Schizoid	6.04 ***	8.84	7.69	2.91	2.28	3.94	2.36	1.15	-	-	-	-	9.93 ***	17.57	5.33	8.57
Depressed	5.40 ***	7.82	6.59	2.91	-	-	-	-	3.17 ***	5.81	4.14	0.83	-	-	-	-
Uncommunicative	7.48 ***	9.86	9.71	4.00	0.89	1.79	0.68	0.46	-	-	-	-	-	-	-	-
Obsessive-Compulsive	7.41 ***	10.20	9.71	3.64	6.05 ***	11.11	6.08	2.76	-	-	-	-	-	-	-	-
Somatic Complaints	4.89 ***	9.86	5.31	1.82	3.27 ***	5.73	3.72	1.38	4.34 ***	8.14	4.79	1.87	1.81	3.60	1.64	0.95
Social-Withdrawal	4.96 ***	6.46	7.33	1.82	-	-	-	-	3.75 ***	6.98	4.14	1.66	-	-	-	-
Hyperactive	2.16	1.70	2.56	2.00	2.08 ***	4.66	1.69	0.69	5.42 ***	7.36	6.97	2.90	-	-	-	-
Aggressive	3.60 ***	5.44	5.31	0.91	2.38 ***	4.66	3.38	0.23	2.59 ***	5.43	3.05	0.62	2.60	3.60	2.46	2.14
Delinquent	5.11 ***	7.82	7.33	1.45	2.58 ***	4.66	3.38	0.69	5.34 ***	8.14	6.75	2.49	2.14	3.15	2.87	1.19
Schizoid-Obsessive	-	-	-	-	-	-	-	-	7.51 ***	12.79	7.84	4.36	-	-	-	-
Sex problems	-	-	-	-	-	-	-	-	3.25 ***	5.43	4.14	1.24	-	-	-	-
Immature-Hyperactive	-	-	-	-	1.39	2.15	2.03	0.46	-	-	-	-	1.92	3.60	2.05	0.95
Hostile Withdrawal	-	-	-	-	3.27 ***	5.02	4.39	1.38	-	-	-	-	-	-	-	-
Anxious-Obsessive	-	-	-	-	-	-	-	-	-	-	-	-	2.26	4.05	2.46	1.19
Depressed-Withdrawal	-	-	-	-	-	-	-	-	-	-	-	-	1.13	1.80	0.41	1.19
Cruel	-	-	-	-	-	-	-	-	4.17 ***	6.98	4.79	2.07	3.39 ***	6.31	3.69	1.67

Note: *** represents significant difference among the left-behind, migrant and local children.

**Table 5:** Regression findings: total problem, internalizing and externalizing behavioral problem.

Variables	Total Problem	Internalizing	Externalizing
	Odds Ratio (OR) Standard Error (S.E.)	OR (S.E.)	OR (S.E.)
Child Category (reference: local children)			
Left-behind children	2.004 *** (0.250)	2.281 *** (0.321)	2.214 *** (0.326)
Migrant children	1.640 *** (0.210)	1.680 *** (0.249)	2.331 *** (0.339)
Gender (reference: boy)	0.894 (0.084)	0.975 (0.104)	0.956 (0.102)
Age (reference: aged 6–11)	0.735 ** (0.073)	0.965 (0.107)	0.965 (0.107)
Health conditions (reference: good physical health)			
Fair physical health	1.391 *** (0.134)	1.501 *** (0.164)	1.423 ** (0.155)
Poor physical health	2.958 *** (0.750)	4.210 *** (1.083)	2.800 *** (0.783)
One-child in family (reference: no)	1.083 (0.108)	1.044 (0.118)	1.216 (0.138)
Household income level (reference: high household income)			
Medium household income	1.954 *** (0.381)	2.396 *** (0.566)	2.934 *** (0.743)
Low household income	2.351 *** (0.474)	2.670 *** (0.651)	2.985 *** (0.779)
Parents marital states (reference: unmarried)	0.612 *** (0.087)	0.958 (0.169)	0.675 * (0.109)
Family conflicts (reference: frequent)	0.657 * (0.111)	0.611 ** (0.114)	0.551 ** (0.102)
Parent alcoholic (reference: no)	1.392 ** (0.159)	1.362 * (0.174)	1.486 ** (0.189)
Maternal education attainment (reference: primary school and below)	0.699 *** (0.075)	0.703 ** (0.083)	0.819 (0.099)
Parent frequently neglect children in the past 6 months (reference: no)	2.438 *** (0.273)	2.225 *** (0.281)	2.471 *** (0.306)
Studied in model-school (reference: no)	0.977 (0.182)	0.942 (0.203)	0.887 (0.190)
Children bullied in school (reference: no)	1.649 *** (0.238)	1.421 * (0.237)	1.156 (0.202)
Residence (reference: urban region)	1.356 * (0.165)	1.353 * (0.189)	1.213 (0.168)
Constant	0.096 ***	0.034 ***	0.035 ***
	(0.030)	(0.012)	(0.013)
N	4479	4479	4479
chi2	265.9	210.1	205.9
Pseudo-R2	0.0775	0.0744	0.0730

Notes: * represents *p* < 0.05, ** *p* < 0.01, *** *p* = 0.000.

**Table 6:** Regression findings: specific syndromes of behavioral problem.

Syndromes		Boys Aged 6–11	Girls Aged 6–11	Boys Aged 12–16	Girls Aged 12–16
		OR (S.E.)	OR (S.E.)	OR (S.E.)	OR (S.E.)
Schizoid	Left-behind	2.700 ** (0.935)	-	3.372 * (2.041)	1.254 (0.370)
	migrant	3.169 *** (1.050)	-	1.538 (1.061)	0.809 (0.329)
Depressed	Left-behind	2.678 ** (0.954)	7.710 *** (4.673)	-	-
	migrant	2.624 ** (0.872)	5.370 ** (3.184)	-	-
Uncommunicative	Left-behind	2.345 ** (0.734)	-	3.187 (3.050)	-
	migrant	2.965 *** (0.849)	-	1.326 (1.616)	-
Obsessive-Compulsive	Left-behind	2.588 ** (0.820)	-	4.152 *** (1.622)	-
	migrant	3.500 *** (1.046)	-	1.725 (0.780)	-
Somatic Complaints	Left-behind	5.923 *** (2.368)	4.089 ** (1.780)	6.133 ** (3.553)	2.568 (1.796)
	migrant	3.164 ** (1.255)	2.581 * (1.163)	1.476 (0.884)	2.756 (2.361)
Social-Withdrawal	Left-behind	3.252 ** (1.363)	4.662 ** (2.208)	-	-
	migrant	5.241 *** (2.027)	2.341 (1.104)	-	-
Hyperactive	Left-behind	0.923 (0.545)	2.675 * (1.068)	7.223 * (5.627)	-
	migrant	0.854 (0.403)	2.518 * (0.929)	1.244 (1.099)	-
Aggressive	Left-behind	5.721 ** (3.072)	9.583 *** (6.499)	23.669 ** (25.874)	1.463 (0.846)
	migrant	7.720 *** (3.957)	5.881 ** (4.027)	10.981 * (12.413)	1.881 (1.223)
Delinquent	Left-behind	4.694 *** (2.031)	3.410 ** (1.371)	6.692 ** (4.757)	1.783 (1.249)
	migrant	6.785 *** (2.840)	2.212 * (0.869)	3.633 (2.814)	4.384 * (3.075)
Schizoid-Obsessive	Left-behind	-	2.737 ** (0.866)	-	-
	migrant	-	2.017 * (0.642)	-	-
Sex problems	Left-behind	-	5.501 ** (3.017)	-	-
	migrant	-	2.597 (1.359)	-	-
Cruel	Left-behind	-	3.018 * (1.311)	-	2.242 (1.224)
	migrant	-	2.590 * (1.109)	-	3.431 (2.202)
Anxious-Obsessive	Left-behind	-	-	-	2.969 (1.980)
	migrant	-	-	-	2.765 (2.040)
Depressed-Withdrawal	Left-behind	-	-	-	0.369 (0.319)
	migrant	-	-	-	0.795 (1.052)
Immature-Hyperactive	Left-behind	-	-	11.187 * (11.612)	2.546 (1.842)
	migrant	-	-	4.534 (4.489)	3.634 (2.950)
Hostile Withdrawal	Left-behind	-	-	4.522 ** (2.630)	-
	migrant	-	-	1.665 (0.973)	-
Covariates		Control	Control	Control	Control

Note: Adjusted for individual factors (gender, age, health condition, only child in family), family circumstances factors (household income level, maternal education attainment, family relationship, drinking habit of parents), school situation factors (key school or not and children being bullied by peers) and residence, reference: local children. * represents *p* < 0.05, ** *p* < 0.01, *** *p* = 0.000.

## References

[1] (2011). The State of the World’s Children. https://www.unicef.org/sowc2011/pdfs/SOWC-2011-Main-Report_EN_02092011.pdf.

[2] Montague M., Enders C., Castro M. (2005). Academic and Behavioral Outcomes for Students at Risk for Emotional and Behavioral Disorders. Behav. Disord..

[3] Jucksch V., Salbach-Andrae H., Lenz K., Goth K., Döpfner M., Poustka F., Freitag C.M., Lehmkuhl G., Lehmkuhl U., Holtmann M. (2011). Severe affective and behavioural dysregulation is associated with significant psychosocial adversity and impairment. J. Child Psychol. Psychiatry.

[4] Marshall S.K., Tilton-Weaver L.C., Stattin H. (2013). Non-suicidal self-injury and depressive symptoms during middle adolescence: A longitudinal analysis. J. Youth Adolesc..

[5] Mehdi T.D., Zahra S., Bahareh P., Azita R., Fatemeh A. (2011). Normative Data and Psychometric Properties of the Child Behavior Checklist and Teacher Rating Form in an Iranian Community Sample. Iran. J. Pediatr..

[6] Giles L.L., Delbello M.P., Stanford K.E., Strakowski S.M. (2007). Child behavior checklist profiles of children and adolescents with and at high risk for developing bipolar disorder. Child Psychiatry Hum. Dev..

[7] Denuwelaere M., Bracke P. (2007). Support and Conflict in the Foster Family and Children’s Well-Being: A Comparison Between Foster and Birth Children. Fam. Relat..

[8] Furstenberg F.F., Nord C.W., Peterson J.L., Zill N. (1982). The Life Course of Children of Divorce: Marital Disruption and Parental Contact. Am. Sociol. Rev..

[9] Glascoe F.P., Maclean W.E., Stone W.L. (1991). The importance of parents’ concerns about their child’s behavior. Clin. Pediatr..

[10] Dodge K.A., Lansford J.E., Burks V.S., Bates J.E., Pettit G.S., Fontaine R., Price J.M. (2003). Peer rejection and social information-processing factors in the development of aggressive behavior problems in children. Child Dev..

[11] Bele S.D., Bodhare T.N., Valsangkar S., Saraf A. (2013). An epidemiological study of emotional and behavioral disorders among children in an urban slum. Psychol. Health Med..

[12] Xin R.E., Zhang Z.X. (1992). Investigate on 24013 city children’s behavioral problems in 26 units of 22 provinces. Shanghai Arch. Psychiatr..

[13] The Sixth National Population Census: Tabulation on the 2010 Population Census of the People’s Republic of China. http://www.stats.gov.cn/tjsj/pcsj/rkpc/6rp/indexch.htm.

[14] Liu J., Liu S., Yan J., Lee E., Mayes L. (2016). The Impact of Life Skills Training on Behavior Problems in Left-Behind Children in Rural China: A Pilot Study. Sch. Psychol. Int..

[15] Wang L., Mesman J. (2015). Child Development in the Face of Rural-to-Urban Migration in China: A Meta-Analytic Review. Perspect. Psychol. Sci..

[16] Achenbach T.M., Rescorla L.A. (2001). Manual for the ASEBA School-Age Forms and Profiles.

[17] Achenbach T.M., Edelbrock C.S. (1981). Behavioral problems and competencies reported by parents of normal and disturbed children aged four through sixteen. Monogr. Soc. Res. Child Dev..

[18] Achenbach T.M. (1983). Manual for the Child Behavior Checklist and Revised Child Behavior Profile.

[19] Duncan S.C., Duncan T.E., Strycker L.A. (2000). Risk and protective factors influencing adolescent problem behavior: A multivariate latent growth curve analysis. Ann. Behav. Med..

[20] Ma X., Yao Y., Zhao X. (2013). Prevalence of behavioral problems and related family functioning among middle school students in an eastern city of China. Asia-Pacif. Psychiatry.

[21] Fan F., Su L., Gill M.K., Birmaher B. (2010). Emotional and behavioral problems of Chinese left-behind children: A preliminary study. Soc. Psychiatry Psychiatr. Epidemiol..

[22] Chang H., Dong X., MacPhail F. (2011). Labor Migration and Time Use Patterns of the Left-behind Children and Elderly in Rural China. World Dev..

[23] He B., Fan J., Liu N., Li H., Wang Y., Williams J., Wong K. (2012). Depression risk of ‘left-behind children’ in rural China. Psychiatry Res..

[24] Zhou C., Sylvia S., Zhang L., Luo R., Yi H., Liu C., Shi Y., Loyalka P., Chu J., Medina A. (2015). China’s Left-Behind Children: Impact Of Parental Migration On Health, Nutrition, And Educational Outcomes. Health Aff..

[25] Zhong B.L., Chen H.H., Zhang J.F., Xu H.M., Fan Y.P. (2010). Detection rate and related factors of behavior problems among children in Wuhan City. Chin. Ment. Health J..

[26] Lu L., Shi Q.J., He H.W., Xu S.J., Chen J.A. (2005). Occurrence of behavioral problems among 4 to 16-year-old children and teenagers in Wuhan city. Chin. J. Clin. Rehabil..

[27] Wang F., Sun Z., Cui Y. (2000). Family environment and behavioral problems in primary and middle school students in rural area of Linyi city. Chin. J. Behav. Med..

[28] Xu W.M., Tang J.L., De W.U., Xu X.Y., Yang L. (2007). Research on Present Situation of Behavior Disorders of Leftbehind Children in the Countryside of Anhui Province. J. Appl. Clin. Pediatr..

[29] Yu X., Dai X., Li Q., Wang L.L., Li L.G. (2014). Logistic Regression Analysis on Behavior Problems and Influence Factors of Left-behind Children in Rural Areas of Ningxia. China J. Health Psychol..

[30] Zhang L., Zhang J.H., Luan J.Z. (2012). Behavior Problem Characteristics of Migrant Children in Baiyun District of Guangzhou. Chin. Gen. Pract..

[31] Achenbach T.M. (1991). Integrative Guide for the 1991 CBCL/4-18, YSR, and TRF Profiles.

[32] Verhulst F.C., Boomsma D.I. (2003). Genetic and environmental contributions to stability and change in children’s internalizing and externalizing problems. J. Am. Acad. Child Adolesc. Psychiatry.

[33] Stormont M. (2002). Externalizing behavior problems in young children: Contributing factors and early intervention. Psychol. Sch..

[34] Narusyte J., Neiderhiser J.M., Andershed A.K., D’Onofrio B.M., Reiss D., Spotts E., Ganiban J., Lichtenstein P. (2011). Parental criticism and externalizing behavior problems in adolescents: The role of environment and genotype–environment correlation. J. Abnorm. Psychol..

[35] Faraone S.V., Perlis R.H., Doyle A.E., Smoller J.W., Goralnick J.J., Holmgren M.A., Sklar P. (2005). Advancing the neuroscience of ADHD: Molecular genetics of attention-deficit/hyperactivity disorder. Biol. Psychiatry.

[36] Rubin K.H., Coplan R.J., Bowker J.C. (2009). Social Withdrawal in Childhood. Ann. Rev. Psychol..

[37] Wang J., Li L., Wu H., Yang X., Wang Y., Wang L. (2014). Agreement between parents and adolescents on emotional and behavioral problems and its associated factors among Chinese school adolescents: A cross-sectional study. BMC Psychiatry.

[38] Tepper P., Liu X., Guo C., Zhai J., Liu T., Li C. (2008). Depressive symptoms in Chinese children and adolescents: Parent, teacher, and self reports. J. Affect. Disord..

[39] Hartley S.L., Sikora D.M., Mccoy R. (2008). Prevalence and risk factors of maladaptive behaviour in young children with Autistic Disorder. J. Intell. Disabil. Res..

[40] Park H., Walton-Moss B. (2012). Parenting style, parenting stress, and children’s health-related behaviors. J. Dev. Behav. Pediatr..

[41] Heflin C.M., Siefert K., Williams D.R. (2005). Food insufficiency and women’s mental health: Findings from a 3-year panel of welfare recipients. Soc. Sci. Med..

[42] Jurma A.M. (2015). Impact of Divorce and Mother’s Psychological Well-Being on Children’s Emotional, Behavioral, and Social Competences. Rev. Cercetare Interv. Soc..

[43] Hu H., Lu S., Huang C.C. (2014). The psychological and behavioral outcomes of migrant and left-behind children in China. Child. Youth Serv. Rev..

[44] Poole-Di S.E., Liu Y.H., Brenner S., Weitzman M. (2010). Adult household smoking is associated with increased child emotional and behavioral problems. J. Dev. Behav. Pediatr..

[45] Leventhal T., Brooksgunn J. (2000). The neighborhoods they live in: The effects of neighborhood residence on child and adolescent outcomes. Psychol. Bull..

